# Pleural Fluid Analysis: Standstill or a Work in Progress?

**DOI:** 10.1155/2012/716235

**Published:** 2012-02-01

**Authors:** T. Hassan, M. Al-Alawi, S. H. Chotirmall, N. G. McElvaney

**Affiliations:** Respiratory Research Division, Educationand Research Center, Royal College of Surgeons in Ireland, Beaumont Hospital, Beaumont Road, Dublin 9, Ireland

## Abstract

Pleural fluid analysis yields important diagnostic information in pleural effusions in combination with clinical history, examination, and radiology. For more than 30 years, the initial and most pragmatic step in this process is to determine whether the fluid is a transudate or an exudate. Light's criteria remain the most robust in separating the transudate-exudate classification which dictates further investigations or management. Recent studies have led to the evaluation and implementation of a number of additional fluid analyses that may improve the diagnostic utility of this method. This paper discusses the current practice and future direction of pleural fluid analysis in determining the aetiology of a pleural effusion. While this has been performed for a few decades, a number of other pleural characteristics are becoming available suggesting that this diagnostic tool is indeed a work in progress.

## 1. Introduction

**Figure 1:**
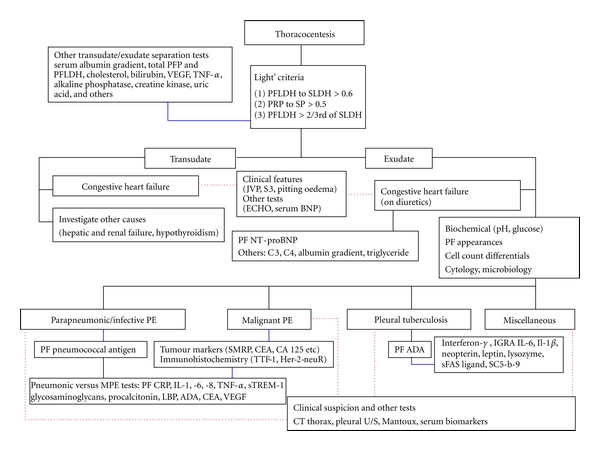
Recommended algorithm for investigation of pleural effusion. The use of Light's criteria is recommended when a thoracocentesis revealed a protein level between 25 and 35 g/L to narrow down the differential diagnosis by determining whether a pleural effusion is transudative or exudative. NT-proBNP should be measured when a suspected cardiac effusion meets the exudative criteria. Determining causes of an exudative effusion is more challenging, and routine test, including biochemical measurement (i.e., pH and glucose), differential cell counts, cytology, and routine microbiology test are diagnostically useful. Pleural fluid pneumococcal antigen has been shown to be superior than urinary antigen to identify bacterial-induced pleural effusion. Tumour marker such as SMRP has a good diagnostic value to diagnose mesothelioma, however, the diagnostic utility of other tumour markers remains limited. Immunocytochemical evaluation of pleural fluid specimen is helpful in labelling different tumour markers. Other biological markers to differentiate parapneumonic/infective and malignant effusion remain elusive, expensive, and not widely available. Testing of pleural fluid ADA is an inexpensive and efficacious method for diagnosing tuberculous effusion, regardless of the patient's immune status. Other tuberculosis-related inflammatory markers are available but are not superior to the latter. (PF: pleural fluid, black continuous line: strongly recommended and routinely practised, blue continuous line: not strongly recommended and not routinely practised, red dotted line: complementary diagnosis with other nonpleural tests.)

Pleural effusions are associated with a number of medical conditions causing fluid accumulation via differing yet synergistic mechanisms including increased pleural membrane permeability, increased pulmonary capillary pressure, decreased oncotic pleural pressure, and lymphatic obstruction. Pleural fluid analysis yields important diagnostic information in most cases of pleural effusions. Standard workup includes determining whether the effusion is transudative or exudative, an important differentiation aiding the physician in narrowing the differential diagnosis (Figure 1). Despite this, several experts propose that such a categorical division represents outdated practice as it does not permit establishing a definitive cause of the effusion. A variety of nonroutine tests may be performed during pleural fluid analysis either as lone or additional diagnostic tools to further determine a definitive cause for an effusion in the appropriate setting. This paper will discuss the current practice and future prospective direction for the use of pleural fluid analysis in determining the aetiology of a pleural effusion in a variety of clinical settings.

## 2. Have We Moved on from Light's Criteria?

The primary aim when investigating a pleural effusion is to establish the correct diagnosis with minimal investigation. Prior to the advent of Light's criteria, most physicians initially determined whether an effusion was transudative or exudative based on the pleural protein level [1]. Serum and fluid albumin gradients of greater than 12 g/L also indicated exudates, however, when used in isolation, these criteria have low sensitivity [2].

Light's criteria have recommended for use when a pleural protein is between 25 and 35 g/L and defines exudative pleural effusions as having either (1) a ratio >0.5 between total pleural and plasma protein, (2) a ratio >0.6 between pleural and plasma lactate dehydrogenase (LDH), and (3) pleural LDH higher than two thirds of the normal serum level. The sensitivity of Light's criteria in identifying exudative pleural effusions is high (98%); however, its ability to exclude transudates remains low. For instance, prospective work by Porcel et al. reported an almost 100% sensitivity for exudates but found that approximately one-fifth of patients with congestive cardiac failure on diuretics also met Light's criteria for an exudate [3]. Despite this deficiency, Light's criteria remain superior to clinical judgement for discriminating between transudates and exudates. A prospective study of *n* = 249 patients directly comparing clinical suspicion to Light's criteria reported that the former was significantly less accurate to the latter (84% versus 93%, *P* < 0.01) (6), illustrating the criteria's importance in routine clinical practice [4].

Most studies to date have focused on making Light's criteria more practical without affecting its discriminatory power. For example, it has been suggested that serum LDH in isolation does not increase the value of the two other criteria components [5]. This is supported by a Brazilian study proposing new criteria to differentiate the two effusion types [6]. By quantifying exclusively total pleural protein and LDH without the need of serum samplings, this study showed a diagnostic yield comparable to that of Light's criteria.

Other studies have additionally generated a number of nonroutine biochemical measurements on pleural fluid that may discriminate transudates from exudates, for instance pleural fluid bilirubin with pleural : serum ratio of >0.6 is suggestive of an exudate with a sensitivity and specificity of 90.6% and 96.2%, respectively [7]. An early case series has demonstrated that pleural fluid cholesterol >60 mg/dL is indicative of an exudative effusion with high sensitivity [8], whilst another study utilizing both LDH and pleural cholesterol measurements revealed similar sensitivity to distinguish between the two types of effusion [9]. Vascular endothelial growth factor (VEGF), an important mediator of angiogenesis and vascular permeability, may be another key mediator and thus marker in the identification of exudates due to the increased pleural endothelial permeability it confers. In one study of *n* = 79 patients, median levels of VEGF in exudates were approximately twice that of transudates [10]. In addition, elevated tumor necrosis factor alpha (TNF-*α*) levels are found in both infectious and malignant pleural effusions, but rarely transudates [11]. Other biochemical parameters that have been examined to aid transudate versus exudate differentiation include alkaline phosphatase, creatine kinase, and uric acid which possessed diagnostic accuracies lower than the traditional Light's criteria [12].

Some of these studies have proposed new and alternative criteria avoiding venepuncture consequently reducing investigations and diagnostic costs. However, these criteria vary in their cut-off points to best discriminate the two forms of effusion. To date, Light's criteria remain robust with diagnostic accuracy of 96% and for now remains the optimal method to separate pleural transudates from exudates.

## 3. Cardiac Pleural Effusion: Extending beyond the Transudate/Exudate Boundary

Cardiac failure remains the most common cause of transudative pleural effusions. A single study has revealed that 28% of cardiac-related pleural effusions were misclassified as exudative due to the use of diuretics [13]. Brain natriuretic peptide (BNP) is a neuroendocrine hormone secreted from the ventricular walls in response to increased pressures and stretch conferred on cardiomyocytes impacting upon the renin-angiotensin system to increase diuresis and vasodilation [14]. BNP is cleaved to NT-proBNP, and detection of the cleaved product in serum has been used to distinguish cardiac failure from primary pulmonary causes of dyspnoea. This marker has been demonstrated to possess diagnostic usefulness during pleural fluid analysis. A study by Long et al. demonstrated that, although levels of pleural BNP have a statistically significant correlation with NT-proBNP, the latter is a far more accurate diagnostic tool during evaluation of cardiac pleural effusions [15]. In addition to high sensitivity and specificity, pleural NT-proBNP is shown to be superior to Light's criteria for the identification of cardiac-based pleural effusions [16]. Meta-analyses have shown that the number of misclassified effusions from application of Light's criteria was significantly reduced with the use of NT-proBNP [17]. However, there is a need for caution as levels of NT-proBNP are physiologically raised in the elderly and renal failure populations requiring further studies to evaluate its role in these groups. Importantly, NT-proBNP measurement is difficult and costly when compared to application of traditional criteria such as the albumin gradient in assessing pleural effusions [18]. Measuring pleural NT-proBNP should therefore be reserved for settings where a suspected cardiac effusion meets exudative criteria but a high index of clinical suspicion remains. Alternative markers have been studied in the assessment of cardiac pleural effusions, for example, normal complement levels (C3 and C4) are reported to have a high negative predictive value in this setting [19].

## 4. The Promise of Biological Pleural Markers to Determine the Aetiology of Pleural Exudates

The most challenging aspect of investigating exudative pleural effusions is differentiating the likelihood of inflammatory parapneumonic versus malignant disease both major causes of exudates in routine clinical practice. Tuberculous effusions are an additional important consideration owing to long-term treatment strategies. Biochemical analyses such as protein, pH, and microbiologic assessment remain the standard investigations during this process. Additionally, cytological examination of suspected malignant pleural effusion (MPE) can result in false-negative rates of up to 40% [20]. The diagnostic yield for cytology, however, depends on the tumour type; highest for ovarian (83%) and less so for breast (78%), lung (57%), and mesothelioma (41%) primaries. Overall, standard testing to determine the underlying cause of exudates has a suboptimal accuracy requiring other parameters such as clinical suspicion and radiology to play an associative role.

### 4.1. Pleural Fluid Appearance

Pleural fluid appearance is a nonspecific and undermined tool in the assessment of a pleural effusion. Prior work has suggested that malignancy is the leading cause of gross bloody effusions (47%) [21] and further confirmed by Porcel and Vives who reported that pleural effusions with significantly higher red blood cell counts occurred in those who subsequently had malignant rather than nonmalignant effusions [22]. In a separate study assessing patients without any prior diagnosis of malignancy, an association between blood-stained effusions and the presence of malignant cells on cytological examination was described [23]. Conversely, a larger retrospective study assessing patients diagnosed with cancer who underwent thoracocentesis revealed no difference between the presence of blood and the ability to predict positive pleural fluid cytology [24]. Therefore, due to low sensitivity and specificity of bloody effusions to indicate malignancy in the setting of an exudate, pleural fluid appearance should not be emphasized as a diagnostic tool to establish MPE as it can be relatively nonspecific.

On the other hand, there are cases where the appearance of pleural fluid may be helpful. The best described setting is one of a milky appearance suggestive of chylothorax or pseudochylothorax that can be differentiated by centrifugation. Although reliable where present, the gross appearance of a chylothorax has been described as nonmilky half the time [25]. Chylothorax may alternatively give the appearance of bile-stained fluid. However, pleural triglyceride analysis is a more definitive test with a level greater than 110 mg/dL reflecting a 99% chance that the fluid is chyle. For infective-related pleural effusions, an anchovy-brown fluid may indicate amoebic liver abscess whilst black fluid suggests *Aspergillus* infection [26].

### 4.2. Cell Count Differential

Differential cell count of pleural fluid may also provide clues to the origin of an exudative pleural effusion. Neutrophilic predominance indicates an acute injury of the pleural surface that may occur in parapneumonic settings, pulmonary embolism, and subphrenic abscesses. In chronic, long standing pleural injury, the fluid becomes populated by lymphocytes. Two-thirds of lymphocytic predominant effusions are the result of malignancy or tuberculosis (TB) [27]. Eosinophilic pleural effusions, defined as a pleural effusion that contains at least 10% eosinophils, most commonly occur during conditions associated with the presence of blood or air in the pleural space such as pneumothorax and malignancy. Interestingly, although eosinophilia is nonspecific and can occur in benign-related effusions, a percentage of pleural eosinophils >40% indicates an extremely low likelihood of malignancy [28].

### 4.3. Markers of Pleural Inflammation

Pleural biological markers have been proposed as an alternative means to determine the cause of exudative pleural effusions. C-reactive protein is an acute-phase reactant widely used as a marker of inflammation and tissue injury. Pleural CRP is found to be higher in benign versus malignant exudates with a sensitivity and specificity of 93.7% and 76.5% for parapneumonic effusion [29, 30]. This finding has been supported by further studies revealing an almost 100% sensitivity for a cut-off value of 5.3 mg/dL to identify parapneumonic versus tuberculous or malignant effusions [31]. Interleukin-8 (IL-8), a proinflammatory cytokine, and CRP together may differentiate complicated from uncomplicated parapneumonic effusions with sensitivities of 84% and 72% and specificities of 82% and 71%, respectively [32]. Interleukin-6 (IL-6), an alternative proinflammatory cytokine induced by lipopolysaccharide (LPS) as a marker of system activation, has also been shown to effectively differentiate infective from malignant effusions with highest levels in tuberculous rather than parapneumonic effusions [33]. In spite of this, elevated IL-6 has also been found in MPE, particularly following pleurodesis. Elevated TNF-*α* is also useful in differentiating tuberculous from malignant effusions [34]. Studies supporting the utility of pleural proinflammatory cytokines during pleural fluid analysis revealed that a low absolute neutrophil count may give a diagnosis of empyema with a sensitivity rate of 78.6% and a specificity rate of 88.4% in the presence of elevated IL-8. Similar findings are reported with IL-1 [35, 36].

Other features of pleural fluid analysis that discriminate infection from inflammation in the complicated versus noncomplicated setting include pleural soluble triggering receptor of myeloid cells-1 (sTREM-1), procalcitonin, and lipopolysaccharide binding protein (LBP) [37]. Pleural pneumococcal antigen assays have been explored and may be more sensitive than the equivalent urinary assays for the establishment of microbial-induced pneumonias [38]. Parapneumonic effusions secondary due to S. pneumoniae are further shown to have positive antigen testing in pleural fluid whilst negative results from concurrent urine sampling.

A prospective study of *n* = 72 patients set out to discriminate exudates with multiple pleural biological parameters including adenosine deaminase (ADA), CRP, carcinoembryogenic antigen (CEA), IL-6, TNF-*α*, and VEGF found ADA and CRP to be the most reliable of the group assessed [39]. ADA concentrations >45 U/L and CRP <4 mg/dL most likely indicated tuberculous effusions, whilst ADA <40 U/L and CRP >6 mg/dL suggested a parapneumonic origin. The latter ADA levels in combination with CRP <4 mg/dL on the other hand, were most likely malignant in origin. In a specialized subgroup of lung transplant recipients, normal or high complement levels within pleural fluid indicate a secondary cause, for example, parapneumonic effusion rather than those attributable to the surgery itself [19].

Despite the large volume of work in-progress, larger and more robust studies are necessitated before we can safely recommend the use of nonroutine and costly biological markers as standard to improve the diagnostic accuracy during the routine workup of an exudative pleural effusion.

### 4.4. Pleural Tuberculosis

Pleural tuberculosis displays important pleural fluid features that significantly contribute to the diagnostic process. Such features preclude the need for invasive investigation such as thoracoscopy or pleural biopsy. Microbiologic assessment remains paramount directly aiding treatment strategy. Microscopic examination of Ziehl-Neelson stained pleural fluid detects acid-fast bacilli in <5% of non-HIV cases [40]. Addition of Lowenstein-Jensen media culture increases this positive yield to approximately 35%. Nucleic acid amplification confers better statistics for diagnosis and has specificity between 90%, and –97%; however, sensitivity may be as low as 60% [41].

ADA is a T-cell (CD4+) metalloenzyme whose presence in high levels within pleural fluid strongly indicated tuberculosis particularly in high prevalence areas. High pleural ADA is also detected in non-TB settings including malignancy, rheumatoid arthritis, systemic lupus erythematosis, and parapneumonic effusions. In view of this, it is important to acknowledge that two ADA isoenzymes exist; ADA 1 and 2 with the latter related in increased levels in the tuberculous setting. In meta-analyses of 63 studies, ADA is reported to have a sensitivity of 92% and a specificity of 90% [42], whilst within the setting of a lymphocytic predominant effusion, ADA >40 U/L is almost exclusively secondary to tuberculosis. Paradoxically, retrospective study of 221 patients has illustrated that ADA levels >250 U/L do not generally occur in tuberculosis related effusions [43]. Such levels are in fact found in patients with empyema or lymphoid-related malignancies. Therefore, whilst the measurement of pleural fluid ADA remains a useful diagnostic tool for tuberculous pleurisy, it should be interpreted in parallel with clinical findings and other traditional methods such as the tuberculin skin test to reach a diagnosis. Such a combination of clinical features with pleural ADA measurement has excellent diagnostic value with a sensitivity and specificity rates of 95% and 97%, respectively. Therefore, experts have recommended measuring ADA levels in low-prevalence areas as a concentration <40 U/L almost exclusively rules out tuberculosis-driven effusions [44].

Measurement of pleural interferon-*γ*, an alternative cytokine derived from lymphocytes, may also be utilized in the diagnosis of tuberculous effusions. However, like ADA, elevated levels of interferon-*γ* are reported in empyema and malignancy. Even though the sensitivity and specificity of this marker is lower than that of ADA, joint sensitivity and specificity utilizing both together increases to 96% and 93%, respectively [45]. A single prospective study of *n* = 63 patients illustrated that interferon-*γ* gamma assays (IGRAs) including the commercially available QuantiFERON-TB Gold and T-SPOT-TB performed poorly compared to interferon-*γ* levels >0.31 IU/mL as a cut-off value [46]. Use of IGRA is currently not recommended due to variability in results when compared to other markers such as ADA that appear superior.

Other biological parameters including pleural IL-6, IL-1*β*, neopterin, leptin, lysozyme, and soluble FAS ligand have extensively been studied in the setting of tuberculous pleural effusions. Neopterin is a pteridine, released by activated macrophages and shown to be elevated in tuberculous effusions [47]. Conversely, leptin, an adipose-derived hormone, has been shown to be reduced to a greater extent in tuberculous effusions when compared to other exudative effusions with a sensitivity and specificity of 82% [48]. Lysozyme also released from activated macrophages similarly have a sensitivity of 85% and specificity 61% for the identification of tuberculous effusions. SC5b-9, a product derived from the binding of C5b-9 complexes to the S protein, is elevated in effusions secondary to TB particularly at a cut-off value of >2 mg/L. Such measurements have also been studied to aid differentiating tuberculous from malignant pleural effusions [49]. Pleural interferon-*γ* inducible 10 k-Da protein (IP-10), interleukin-12 p40, and matrix metalloproteinase (MMP) levels [50–52] are additional markers elevated in pleural fluid from tuberculous effusions compared with malignant and other benign settings. Despite this, their significant variability in sensitivity and specificity coupled with costs for routine use preclude the introduction of such measures into routine clinical practice. What is probably most feasible is a combination of these tests used synergistically at a reduced cost. For example, a study has shown that a combination assay including ADA, interferon-*γ*, and nucleic acid amplification for TB will have superior sensitivity and specificity as compared to a single test alone and offers a future promise in the workup of tuberculous effusions [53].

### 4.5. Tumour Markers

Nodularity, pleural and diaphragmatic thickening are highly indicative of malignant pleural disease with a positive predictive value of 100%. Despite this, such features are not always present and pleural fluid cytology thus plays a crucial role in the diagnosis of malignancy. MPEs are positive in 40–60% of cases [54], and it is common practice to require a large volume (>500 mLs) of fluid to reach a diagnosis by cytology. Interestingly, a recent prospective study on *n* = 121 thoracocentesis showed that diagnostic accuracy was dependent on the volume of pleural fluid obtained with a recommendation of >150 mL, whilst another prospective study examining *n* = 44 patients concluded that samples >50 mL similarly did not increase diagnostic yields [55, 56]. Work on noninvasive tumour biomarkers remain ongoing and if successful may avoid invasive investigation such as pleural biopsy or thoracoscopy.

Well-described tumour markers such as carcinoembryonic antigen (CEA), cancer antigen 125 (CA 125), 15-3 (CA 15-3), and neuron-specific enolase/cytokine fragment (CYFRA) 21-1 have limited usefulness in the routine workup of a suspected malignant pleural effusion. CA 125 is a well-described tumour marker implicated in ovarian malignancy and has reported high levels in pleural fluid compared to serum in this setting [57]. High pleural CA 125, however, is also observed in squamous and adenocarcinomatous malignant effusions and may have a prognostic role [58]. Pleural CEA was one of the first markers to be evaluated in lung cancer. It is overexpressed in metastatic adenocarcinoma possessing prognostic relevance in terms of median survival and treatment response [59]. Although CEA has been shown to be specific, sensitivity remains low (<50%) with variable cut-off values precluding routine use. Metastatic breast cancer is another common cause for malignant pleural effusions with CA 15-3 used for both diagnosis and therapeutic monitoring [60]. CYFRA 21-1, a cytokeratin tumour marker has both diagnostic and prognostic roles in nonsmall cell lung cancer [60]. However, due to relatively poor sensitivity for a single test, use of a combination of tumour markers such as CA 125 and CYFRA 21-1 for adenocarcinoma and CEA, CA 15-3, and CYFRA 21-1 for squamous cell cancer of the lung has been recommended. In a study involving *n* = 243 and *n* = 173 patients with malignant and benign effusions, respectively, selected cut-off values had to be 100% specific to classify correctly 54% of the malignant effusions [61]. Discriminating pleural fluid cut-off values were generally higher than those found in serum, a finding that does not justify the routine use of measuring classic tumour markers in the workup of pleural effusions. Despite this, one particular study has in fact demonstrated that in cases of suspicious MPE and negative cytology and in the absence of an obvious primary source that the measurement of tumour markers may be helpful as an alternate diagnostic tool [62].

The diagnosis of a mesothelioma-related pleural effusion remains difficult as few studies have reported markers with a high positive predictive value. Increased levels of pleural CA-15-3, hyaluronic acid, and spliced forms of CD44, such as exon v6 (CD44v6), have been reported; however, a discrepancy exists in the literature for CYFRA 21-1 to differentiate between mesothelioma and other pleural malignancies [63]. Alternatively, mesothelin, a cell surface glycoprotein that may be cleaved into the soluble mesothelin related protein (SMRP), is a moderately sensitive but highly specific marker in serum studies [64]. Studies also support the use of pleural fluid levels of mesothelin with 98% specificity and 67% sensitivity in mesothelioma compared to benign effusions [65, 66]. Pleural SMRP measurement also diagnosed mesothelioma more reliably than cytological examination alone [65, 67]. Consequently, such a measure could be considered for patients with undiagnosed pleural effusions, particularly if mesothelioma is a concern.

Immunocytochemistry may also be performed on cytological pleural fluid specimens. For example, several mesothelial markers such as calretinin, keratin 5/6, and WT-1 protein may be used in conjunction with carcinoma markers such as thyroid transcription factor-1 (TTF-1), CEA, and B72.3. These may be used to effectively discriminate epithelial mesothelioma from adenocarcinoma [68]. Whilst 80% of lung adenocarcinoma exhibits TTF-1 positivity, a positive TTF-1 stain in pleural fluid without an established primary cause may alternatively suggest primary non-small cell lung cancer [68]. In the setting of breast malignancy, use of pleural Her-2-neu receptor positivity has diagnostic and therapeutic implications [69]. Although useful in particular settings, the weaknesses associated with the routine use of measuring tumour biomarkers in pleural fluid should be recognized by clinicians.

### 4.6. Rheumatological-Related Pleural Effusions

Rheumatological-related pleural effusions usually have pleural biochemical characteristics such as protein, pH, and glucose similar to other noninfective causes of exudates. Serum rheumatoid factor (RF) and antinuclear antibody (ANA) are more sensitive and specific for the diagnosis of rheumatoid arthritis (RA) and systematic lupus erythematosis (SLE); however, their measurement in pleural fluid does not possess the same diagnostic accuracy for disease-related pleural effusions. RF titres may be measured in pleural fluid and are often >1 : 320 in rheumatoid pleuritis. Despite this finding, this measurement is rarely practiced in the clinical setting as the pleural levels often reflect serum values. One particular study confirmed this fact as it reported no additional diagnostic value above that of serum analysis alone [70]. Cytological assessment looking for “ragocyte” cells that consist of white blood cells with phagocytic intracellular inclusions is described in rheumatoid pleuritis but generally has low specificity. Pleural ANA is additionally not specific to SLE and may occur in malignancies. Interestingly, however, one study has suggested that pleural ANA measurement possesses good negative predictive value for SLE pleuritis and may be useful in this context [71]. Conversely, an alternative study of *n* = 266 patients has shown no additional value in measuring pleural ANA in the setting of SLE pleuritis [72]. Pleural antidouble stranded DNA (dsDNA) also has good negative predictive value, whilst complement activation of pleural fluid in both RA and SLE has also been evaluated but is not routinely practiced in the clinical setting due to its low specificity [73].

## 5. Conclusion

Since the introduction of Light's criteria in the 1970s, other proposed criteria and recommendations to overcome some of its drawbacks have been suggested, however, Light's criteria have remained biochemically the most robust for differentiating exudates from transudates. This allows easier diagnosis for an underlying cause of a pleural effusion, avoiding unnecessary investigation. Nevertheless, most transudates are in fact secondary to congestive heart failure where clinical judgement and disease-specific markers such as NT-proBNP have been proven to be superior. Other useful disease-specific markers include ADA in the diagnosis of tuberculous effusions. Discriminating malignant and benign pleural effusions in the setting of an exudate remains a challenge. While there is no substitute to the histologic demonstration of malignancy, the role of tumour markers may emerge to have a larger contribution in the future as a complementary tool in the setting of MPE particularly when considering the invasiveness and lack of universal accessibility to thoracoscopy. Although workup of pleural effusions is a mix of both old and new measures, novel technologies such as global gene profiling and proteomics enable the identification of “fingerprints” for disease-specific markers that will undoubtedly improve our approach in the diagnosis for a definitive cause of a particular pleural effusion. Such future improvements do illustrate major advances from the simplistic transudate-exudate separation and do suggest that pleural fluid analysis is in fact a work in progress.
